# Measuring recovery-oriented rehabilitation language in clinical documentation to enhance recovery-oriented practice

**DOI:** 10.1192/bjo.2023.14

**Published:** 2023-02-15

**Authors:** Veronica De Monte, Angus Veitch, Frances Dark, Carla Meurk, Marianne Wyder, Maddison Wheeler, Kylie Carney, Stephen Parker, Steve Kisely, Dan Siskind

**Affiliations:** Mobile Intensive Rehabilitation Team, Metro South Addiction and Mental Health Service, Woolloongabba, Queenland, Australia; Department of Media and Communications, Swinburne University of Technology, Melbourne, Victoria, Australia; Mobile Intensive Rehabilitation Team, Metro South Addiction and Mental Health Service, Woolloongabba, Queenland, Australia; and Faculty of Medicine, University of Queensland, Brisbane, Queensland, Australia; Faculty of Medicine, University of Queensland, Brisbane, Queensland, Australia; and Queensland Centre for Mental Health Research, The Park, Wacol, Queensland, Australia; Research and Learning Network, Metro South Addiction and Mental Health Service, Mt Gravatt, Queensland, Australia; Faculty of Medicine, University of Queensland, Brisbane, Queensland, Australia; and Research and Learning Network, Metro South Addiction and Mental Health Service, Mt Gravatt, Queensland, Australia; Mobile Intensive Rehabilitation Team, Metro South Addiction and Mental Health Service, Woolloongabba, Queenland, Australia; Faculty of Medicine, University of Queensland, Brisbane, Queensland, Australia; and Queensland Centre for Mental Health Research, The Park, Wacol, Queensland, Australia

**Keywords:** Recovery, rehabilitation, language, documentation, community mental health teams

## Abstract

**Background:**

Mental health services are encouraged to use language consistent with principles of recovery-oriented practice. This study presents a novel approach for identifying whether clinical documentation contains recovery-oriented rehabilitation language, and evaluates an intervention to improve the language used within a community-based rehabilitation team.

**Aims:**

This is a pilot study of training to enhance recovery-oriented rehabilitation language written in care review summaries, as measured through a text-based analysis of language used in mental health clinical documentation.

**Method:**

Eleven case managers participated in a programme that included instruction in recovery-oriented rehabilitation principles. Outcomes were measured with automated textual analysis of clinical documentation, using a custom-built dictionary of rehabilitation-consistent, person-centred and pejorative terms. Automated analyses were run on Konstanz Information Miner (KNIME), an open-source data analytics platform. Differences in the frequency of term categories in 50 pre-training and 77 post-training documents were analysed with inferential statistics.

**Results:**

The average percentage of sentences with recovery-oriented rehabilitation terms increased from 37% before the intervention to 48% afterward, a relative increase of 28% (*P* < 0.001). There was no significant change in use of person-centred or pejorative terms, possibly because of a relatively high frequency of person-centred language (22% of sentences) and low use of pejorative language (2.3% of sentences) at baseline.

**Conclusions:**

This computer-driven textual analysis method identified improvements in recovery-oriented rehabilitation language following training. Our study suggests that brief interventions can affect the language of clinical documentation, and that automated text-analysis may represent a promising approach for rapidly assessing recovery-oriented rehabilitation language in mental health services.

Person-centred and recovery-oriented practices are now a central tenet of mental health service delivery, both within Australia and internationally.^1,2^ Compared with treatment-oriented clinical mental health services, rehabilitation services place differential emphasis on collaborative work done with (rather than to) a person to enhance cognitive, social, emotional and occupational functioning.^3^ It is therefore important that clinical documentation in rehabilitation services adequately describes these interventions, records language that avoids dehumanising patients^4,5^ and therefore reflects recovery-oriented practices and attitudes to all audiences, meaning that ensuring the language recorded is recovery-oriented in nature is important and relevant regardless of whether the documentation is shared with patients. Furthermore, the type of language adopted by mental health services, including that recorded in clinical documentation, has been found to be a mediator in the behaviours and attitudes of mental health clinicians.^3^ Despite this, the documentation produced by mental health clinicians, and the degree to which it is recovery-oriented, has received little attention in the literature.

In a preliminary exploration of the relationship between language and recovery-oriented practice, Kemp and Howard concluded that language was a ‘gap in the research of the recovery approach’, and that more systematic research in specific mental health settings was needed.^4^ Therefore, the current study implemented and evaluated an intervention to increase the amount of recovery-oriented rehabilitation and person-centred language, and decrease the amount of pejorative language, written down in care review summaries in a public mental health intensive rehabilitation service. The setting was the Metropolitan region of Queensland, Australia. To evaluate the hypothesised changes in language from pre- to post-intervention, an automated, computational content analysis approach was developed based on the generation of a custom-built dictionary of recovery-oriented rehabilitation-consistent, person-centred and pejorative terms. As clinical information is now increasingly available via electronic health records, the use of automated approaches to audit clinical information has also increased because of the savings in time and resources compared with traditional qualitative analysis.^6–8^ This data resource is relatively underutilised and provides an opportunity for mental health research.^8^ Table 1 gives examples of each language type.

**Table 1:** Examples of the different types of language evaluated in this study^9,10^

	Language type		
	Recovery-oriented rehabilitation (refers to the development of skills and support needed to achieve one's goals through promotion of strengths and partnerships)	Person-centred (places the individual at the centre and reflects unconditional positive regard)	Pejorative (expresses contempt or disapproval, or intends to disparage or belittle)
Example	Emphasises abilities/strengths, e.g. ‘X has strengths/skills/passions in … ’ Conveys hope and optimism in abilities and promotes recovery, e.g. ‘What is important to you? What are you looking forward to?’ Describes goals and steps taken toward achieving these Indicates formulation and collaboration	Use of the person's name Describes behaviour rather than labels, e.g. ‘X is worried that … ’ (instead of delusional) Put people first, e.g. ‘X has a mental health condition/schizophrenia’ Genuine, honest, easy-to-follow language The person's own language Validating of the person's experience Indicates that the person has understood and been given the opportunity to discuss information	Condescending Patronising Tokenistic Intimidating Discriminating Judgemental Sensationalises mental illness Portrays successful people with mental illness as rare
Non-example	Emphasis on limitations or what is wrong with the person Promotes pessimism and illness model Emphasis on interventions done to rather than with the person Emphasis on failures of the past Indicates a treatment plan that was devised without consulting the person	Overuse of terms such as patient/client/consumer Labelling, e.g. ‘X is mentally ill/ delusional/paranoid/schizophrenic/chronic/non-compliant’ Jargon or specialist language Indicates assumption that information is understood, agreed with and seen as relevant Disputes the person's perception of events Indicates assumption that you know what is best for the person	Respectful Non-judgemental Inclusive

## Hypotheses

We hypothesised that following the delivery of an intervention to promote increased recording of rehabilitation-focused language in documentation, care review summary documents would include more recovery-oriented rehabilitation and person-centred language. Several specific primary hypotheses were tested in this study.

- Care review summaries after the intervention will show increased rates of recovery-oriented rehabilitation words, phrases and themes.
- The rate of person-centred words, phrases and themes that are not also recovery-oriented rehabilitation words, phrases or themes will increase following the intervention.
- There will be a decrease in words, phrases or themes that directly contravene person-centred principles, or are derogatory in nature, termed pejorative language.

## Method

### Ethics

Ethics approval was obtained before study commencement, from the Metro South Human Research Ethics Committee (reference number: HREC/18/QPAH/24), and all participation in this study was based on voluntary informed consent.

The authors assert that all procedures contributing to this work comply with the ethical standards of the relevant national and institutional committees on human experimentation and with the Helsinki Declaration of 1975, as revised in 2008. All procedures involving participants were approved by Metro South Human Research Ethics Committee. All participants provided written informed consent to participate in this study.

### Study context

The pilot intervention was trialled in a community-based public mental health service model that provides intensive care for people with severe mental illnesses (predominantly schizophrenia) to work on their recovery goals (e.g. live independently, return to work/study, access the community). It follows an assertive community treatment model, but with an enhanced emphasis on psychosocial rehabilitation. Beyond intensive case management and medication support, a range of evidence-based psychosocial interventions are available (e.g. cognitive–behavioural therapy, cognitive remediation, social cognitive interventions and vocational rehabilitation support). Previous evaluation has found engagement with the service is associated with functional improvement for consumers.^11^

A critical component of the service model is the availability of intensive case management, with case management roles being filled by a range of clinical disciplines (occupational therapy, nursing, psychology and social work). It is an expectation that case managers will complete care review summaries every 3 months. These summaries provide an overview of a person's progress over the previous 3 months, and outlines their goals and plan for moving forward. These summaries are generally produced as a record for the service and, at the time of this study, were not commonly shared with patients. However, there were concerns that the language used by case managers in these care review summaries was not recovery-oriented rehabilitation language and did not reflect actual collaboration between the case manager and the consumer. This was the reason for the present study.

### Intervention

A training programme was developed to improve the use of recovery-oriented rehabilitation language in care review summaries. The intervention provided case managers with the opportunity to reflect on the use of the language documented in their care reviews. The group intervention involved two in-service presentations by the team neuropsychologist that were delivered 1 week apart to the rehabilitation team leader, consultant psychiatrist, psychiatric registrar and case managers. All clinicians were then mentored in a one-on-one session by the neuropsychologist, which included feedback on their care reviews. Table 2 provides a brief summary of the training provided.

**Table 2:** Outline of intervention training

Intervention	
Group training session 1	
Aims	Education on recovery-oriented language, formulation and treatment planning in rehabilitation
Resources	Training session 1 PowerPoint presentation Copies of the following resources for each mental health practitioner: Is diagnosis enough to guide interventions in mental health? Using case formulation in clinical practice paper^12^ Guide to appropriate language in mental health services^9^ Feedback form for training session 1
Content	Rationale for the training and evaluation Rehabilitation What is recovery versus rehabilitation Definitions of recovery and rehabilitation Importance of rehabilitation Formulation in rehabilitation Functions of formulation Importance of immediate, ongoing and collaborative formulation 5 Ps of formulation Using formulation to plan rehabilitation
Process	30 min presentation and discussion Questions encouraged throughout the training rather than waiting until the end Group discussions: Recovery versus rehabilitation Group formulation of a current consumer Allocated time for feedback of the presentation by clinicians
Group training session 2	
Aims	Education on how to document the concepts outlined in group training session 1, using recovery-oriented rehabilitation language
Resources	Training session 2 PowerPoint presentation Guide to appropriate language in mental health services^9^ Copies of the following resources for each mental health practitioner: Recovery-Oriented System Indicators (ROSI)^13^ Recovery Self-Assessment (RSA)^14^ Recovery and Independent Living PEG Advisory Paper 9^15^ Feedback form for training session 2 Feedback form for training session 2
Content	Recap of content from training session 1 Recovery-oriented rehabilitation language What it is Importance of it Purpose of it Who the audience might be How to document using recovery-oriented rehabilitation language Aspects to consider/include Useful tools to assist use of recovery-oriented rehabilitation language Importance of person-centred language
Process	30 min presentation and discussion Questions encouraged throughout the training rather than waiting until the end Group discussions: Why recovery-oriented rehabilitation language is a good idea Who are you writing for and what do they want to know Allocated time for feedback of the presentation by clinicians
Individual training session	
Aims	Provide one-on-one support to clinicians to develop specific formulations and documentation, as well as time for clinicians to ask questions and refine formulation and recovery-oriented language skills
Resources	Example case review to discuss Is diagnosis enough to guide interventions in mental health? Using case formulation in clinical practice paper^12^ Guide to appropriate language in mental health services: New South Wales Government
Content	Guided discussion of the following points: Importance of recovery-oriented rehabilitation language to the clinician Use of formulation, in particular the presenting problem and the perpetuating factors The work completed with the consumer during this care period, using rehabilitation-oriented language, including: What skills did the consumer develop? What supports were put in place/enhanced? How are the skills and supports working together to aid recovery? How have abilities increased? What partnerships were formed/ quality of partnerships? What core/specific partnership tasks were undertaken? What were the outcomes measures that were used? Outline of the recovery plan, using rehabilitation-oriented language, for the upcoming care period based on formulation and work already completed Use of patient-centred language throughout Refer to the appendix of the Guide to appropriate language in mental health services (NSW Government) for rephrasing into patient-centred language^9^
Process	30–60 min guided individual, face-to-face discussion of one case review Ensure the clinician has opportunity and encouragement to ask questions

### Recruitment and data collection

The data for the evaluation were de-identified care review summaries completed by the clinicians. All 11 case managers in the team consented to participate. To evaluate the effectiveness of the intervention, we conducted an uncontrolled pre–post textual analysis of the care review summaries. For this, care review summaries completed by consenting clinicians before delivery of the pilot intervention were the ‘pre-sample’ data-set. This was then compared with care review summaries completed after the delivery of the intervention (the ‘post-sample’ data-set). The time frame between completion of the pre-sample data-set and the completion of the post-sample data-set was approximately 5 months.

### Definitions

The Psychiatric Rehabilitation Association defines recovery as a process through which a person with mental health difficulties gains ‘a sense of meaning, a positive identity, fulfilling relationships, the role of citizen and community member, the capacity to cope with adversity and recognition of the gifts and lessons learned through the recovery struggle’.^10^ Rehabilitation ‘refers to the development of skills and supports needed to achieve one's goals … focuses on increasing ability and builds on a person's strengths to facilitate success in meeting the person's own goals … Rehabilitation promotes a partnership (between person and clinician)’.^10^ In other words, recovery refers to the person's individual experience and journey through mental health difficulties to develop and pursue their goals, whereas rehabilitation is a vehicle for recovery that can involve both the person and clinician. Given this distinction, and that the aim of this study was for care review summaries to better reflect the work undertaken by clinicians in collaboration with patients, we chose to use the term ‘recovery-oriented rehabilitation language’ instead of simply ‘recovery-focused’ or ‘recovery-oriented’.

Recovery-oriented rehabilitation language also encompasses person-centred language as it places the individual at the centre of the service and reflects unconditional positive regard for the person being spoken with or written about.^1,10^ In this study, we conceptualised person-centred language as being a broader term that denotes respect for the person, first and foremost, and is non-judgemental, clear and understandable, and carries a sense of commitment, hope and opportunity.^9,10^

**Fig. 1:**
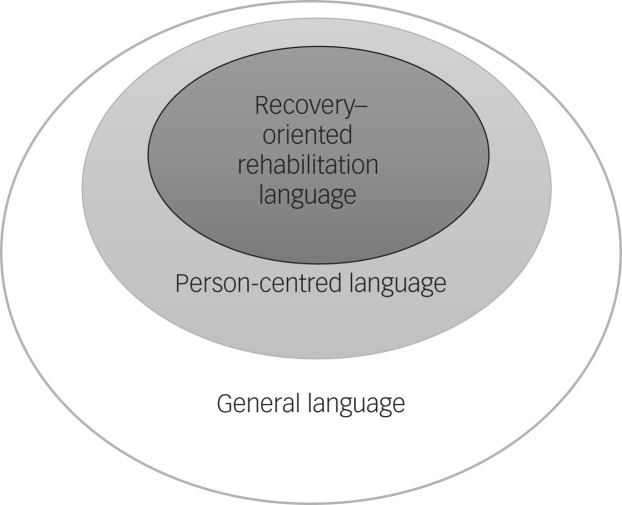
Conceptual framework for the different types of language evaluated in this study.

Given these principles, particularly that of conveying hope, commitment and opportunity, recovery-oriented rehabilitation language is necessarily person-centred by nature. However person-centred language is not necessarily rehabilitation-focused. For example, the use of the person's name instead of the label patient/client/consumer is considered to be highly person-centred, but does not necessarily convey a rehabilitation focus. Similarly, shifting language from declarative and impersonal medicalised statements, such as the statement ‘the patient is delusional’, to person-centred language that understands these experiences from the individual's perspective and the distress it causes (e.g. in terms of ‘the person is worried that … ’), is an important part of a person-centred approach but does not convey information about how recovery may be facilitated through rehabilitation.^9^ Figure 1 summarises the relationship between recovery-oriented rehabilitation language, person-centred language and language more generally, or neutral language. Table 1 provides examples of recovery-oriented rehabilitation and person-centred language, as well as pejorative language.

### Development of a custom-built dictionary

To evaluate changes in language from pre- to post-intervention, we compiled a dictionary of terms pertaining to each language category (recovery-oriented rehabilitation, person-centred and pejorative). Traditionally, the coding of text in this way is performed manually by multiple readers, with reference to established words or phrases that indicate the language being analysed. Manual coding of text allows for nuanced and contextually informed judgements about the presence or absence of textual features. Although this approach is labour-intensive and established content analysis dictionaries can allow for more automated coding, such dictionaries are often not applicable outside of the contexts or domains in which they are developed. As far as we are aware, no dictionary has been published that categorises recovery-oriented rehabilitation language. Therefore, it was decided that straightforward statistical techniques in combination with selective manual coding would be used, and dictionary terms would be defined as indicators of a given language type, rather than complete instances or partial components. It was also decided that the presence of language types would be measured at the level of whole sentences rather than by individual terms, to allow individual sentences to be coded with multiple categories, define language type by multiple words in combination or sequence, and reduce the risk of double counting a language instance that contains two or more dictionary terms. To allow the dictionary to encode grammatically or semantically distinct occurrences of a given word, sequences of up to three words long (known as bigrams and trigrams, or more generically, ngrams) were included in addition to single words; for example, the dictionary terms ‘CNAME will’ and ‘CNAME was’, where CNAME denotes the consumer's name.

An initial set of dictionary terms was identified with the New South Wales Mental Health Coordinating Council guidelines for use of person-centred language.^9^ The study team identified terms from this document that were deemed to be rehabilitation-oriented, person-centred or non-person-centred. To supplement this, we analysed 728 short segments of manually classified text sampled from all care review summaries in the data-set. This coding, in which a language category was assigned to each text segment, was performed by a member of the research team, and reviewed by a second member. Where the second member disagreed with the original member's coding, the segment was discussed with the wider research team. Agreement on coding was reached 100% of the time by using this method. We searched for sequences of one, two or three words whose occurrence showed a strong relationship with any of the language categories. These terms were then manually inspected to confirm that they were reliable indicators of the associated categories.

Having compiled a first draft of the dictionary from these two sources, we expanded and refined the listed terms by examining their sentence-level co-occurrences with the dictionary categories and with unclassified words in the data-set. To do this, we used a measure called normalised pointwise mutual information, which essentially compares the observed co-occurrence of two features with their expected co-occurrence, given how often they each occur independently. The result is a number from –1 to 1, which can be interpreted in much the same way as a correlation coefficient. If, by this measure, a dictionary term had a weak association with its own category, or a stronger association with a different category, then its classification was reviewed. If an unclassified term had a strong association with a dictionary category, it was considered for inclusion in the dictionary. A total of 130 dictionary terms were identified (Supplementary Table 1 available at https://doi.org/10.1192/bjo.2023.14).

### Data analysis

Care review summaries were de-identified (excluding the pre-populated fields and header), processed and analysed computationally with Konstanz Information Miner (KNIME 4.7.0 for Windows; KNIME AG, Zurich, Switzerland; https://www.knime.com/), an open-source, visually oriented data analytics platform that includes specialised text-processing functionality. To achieve a more consistent sentence length, we split sentences longer than 30 words into strings no longer than 20 words, splitting around punctuation marks such as periods and commas wherever possible. In addition, we merged sentences shorter than five words into adjacent sentences. Processed in this manner, the data-set contained 6740 sentences with an average length of 16 words. We measured the presence of a given language category within a document by calculating the percentage of sentences in which the language category appeared. To assess the impact of the intervention, we calculated and compared average document-level percentages before and after the intervention. To assess qualitative changes in language use, we compared the presence of individual dictionary terms before and after the intervention. As with the language categories, the metric we used was percentage of sentences per document in which each term occurred.

*t*-Tests compared the percentage of sentences containing recovery-oriented rehabilitation, person-centred and pejorative language pre- and post-intervention, and the percentage of sentences containing specific terms pre- and post-intervention. As this was a pilot study to inform further research, we did not conduct power calculations.

As three of the researchers in this study have clinical supervisory roles with the participants, all data were de-identified and were not re-identifiable. All data collection and analyses were conducted by researchers independent of the Mobile Intensive Rehabilitation Team within the Metro South Addiction and Mental Health Service. Researchers associated with Mobile Intensive Rehabilitation Team did not have access to any case manager- or consumer-level data.

## Results

A total of 50 pre-intervention and 77 post-intervention care review summaries were included in analyses. Each set of care review summaries consisted of the most recent care review summary written by the case managers of all patients treated by the team at that time point (with the exception of those written by the team neuropsychologist).

A significant increase in the use of recovery-oriented rehabilitation language was found, with the average percentage of sentences in which recovery-oriented rehabilitation terms occurred increasing from 37% before the intervention to 48% afterward, translating to a relative increase of 28% (*P* < 0.001). There was no significant change in person-centred language. Pejorative language appeared in a very small fraction of sentences both before and after the intervention. The prevalence of pejorative language decreased, but not to a degree that was statistically significant. Table 3 outlines changes in the frequency of use of the different language categories pre- and post-intervention.

**Table 3:** Prominence of language categories (average percentage of sentences that contain the category) before and after the intervention

Category	Average percentage of sentences with terms pre-intervention	Average percentage of sentences with terms post-intervention	Ratio	*P*-value
Recovery-oriented rehabilitation	37% (s.d. = 12)	48% (s.d. = −12)	1.28	<0.001
Person-centred	22% (s.d. = 6)	22% (s.d. = 7)	−1.01	0.808
Pejorative	2.3% (s.d. = 3.0)	1.6% (s.d. = 2.3)	−1.40	0.176

Table 4 summarises the most significant changes in term usage within the recovery-oriented rehabilitation and person-centred language categories. It shows the average percentage of sentences in which each term occurred before and after the intervention. The change in percentage points reflects how much a term's usage increased or decreased in relative terms.

**Table 4:** Changes in term frequency (average percentage of sentences) before and after the intervention

Category	Term	Before intervention	Before intervention s.d.	After intervention	After intervention s.d.	Change (% points)	Ratio	*P*-value
Recovery-oriented language	Well-being	0.04%	0.23	1.22%	2.19	1.17	27.53	0.001
	CNAME will	0.07%	0.32	1.25%	3.23	1.18	18.19	0.013
	Challenges	0.08%	0.29	1.13%	1.91	1.05	14.89	0.001
	Connections	0.04%	0.19	0.52%	1.06	0.49	14.02	0.002
	Vocational	0.16%	0.55	1.01%	2.28	0.85	6.33	0.012
	Perpetuating	0.07%	0.32	0.43%	0.84	0.36	6.30	0.005
	Care plan	0.06%	0.29	0.33%	0.74	0.27	5.94	0.016
	Associated with	0.24%	0.61	1.21%	3.23	0.97	4.98	0.041
	Domestic	0.29%	0.75	1.39%	2.51	1.11	4.87	0.004
	Metabolic monitoring	0.10%	0.45	0.46%	0.83	0.36	4.60	0.006
	Participating	0.10%	0.38	0.41%	1.07	0.32	4.27	0.049
	Factors	0.46%	1.42	1.67%	3.85	1.21	3.62	0.037
	Maintaining	0.59%	1.54	2.11%	3.27	1.52	3.60	0.003
	Partnership	0.22%	0.63	0.76%	1.14	0.54	3.43	0.003
	Will	1.13%	1.52	3.65%	5.63	2.51	3.22	0.003
	In next	0.21%	0.65	0.67%	1.41	0.46	3.20	0.033
	Difficulties	0.80%	1.82	2.55%	2.54	1.75	3.19	0.001
	Rehab	0.50%	1.24	1.48%	2.81	0.99	2.99	0.022
	Engages	0.17%	0.55	0.51%	0.97	0.33	2.94	0.030
	Opportunities	0.30%	0.88	0.75%	1.35	0.45	2.50	0.040
	Engaged	1.34%	1.55	0.75%	1.31	−0.59	0.56	0.022
	Offered	0.50%	1.02	0.17%	0.55	−0.33	0.34	0.019
	Demonstrated	0.53%	1.17	0.16%	0.55	−0.37	0.30	0.018
	Group	1.38%	2.29	0.41%	1.09	−0.98	0.30	0.002
	Meals	0.52%	1.05	0.14%	0.53	−0.38	0.28	0.009
	Plans	0.58%	1.09	0.14%	0.49	−0.44	0.24	0.003
	Encouragement	0.41%	0.94	0.09%	0.36	−0.32	0.22	0.009
	Swimming	0.41%	0.88	0.08%	0.40	−0.34	0.19	0.004
	Clean	0.35%	0.70	0.06%	0.32	−0.29	0.18	0.002
	Living skills	0.58%	1.22	0.03%	0.21	−0.55	0.05	0.001
Person-centred language	Enduring	0.02%	0.17	0.84%	1.65	0.82	43.18	0.001
	Predisposed	0.06%	0.44	0.58%	1.19	0.52	9.12	0.005
	Establishing	0.12%	0.41	0.61%	1.24	0.49	5.22	0.009
	Understanding	0.26%	0.84	1.06%	1.91	0.80	4.04	0.007
	Inability	0.12%	0.43	0.38%	0.83	0.26	3.22	0.042
	Continues to	2.13%	2.44	1.26%	1.66	−0.87	0.59	0.019
	CNAME was	3.99%	3.70	2.09%	2.69	−1.90	0.52	0.001
	CNAME had	1.27%	1.60	0.58%	1.34	−0.69	0.46	0.010

Only terms with statistically significant change are listed. Some terms when taken out of context do not immediately appear to correspond to recovery-oriented rehabilitation language, but were related to recovery goals or approaches when in context. For example, the term ‘metabolic monitoring’ related to patients working to improve their physical health with the help of monitoring by clinicians. CNAME, consumer's name de-identified.

The term accounting for the greatest relative increase in the recovery-oriented rehabilitation category is ‘well-being’, followed by ‘CNAME will’ (where CNAME is a placeholder for the consumer's name). Although the use of recovery-oriented rehabilitation language increased overall, some specific terms decreased following the intervention, albeit only modestly.

Although the use of person-centred language did not change overall, the use of certain terms in this category did change. The term that increased the most was ‘enduring’, which is typically seen as being more person-centred than a term such as ‘chronic’.

The use of some specific terms that are not recovery-oriented rehabilitation language, such as ‘not been able’, ‘is not able’ and ‘CNAME was’ decreased from pre-intervention to post-intervention.

## Discussion

This study was an evaluation of a training programme designed to promote the use of recovery-oriented rehabilitation language in clinical documentation in a rehabilitation-oriented community mental health team. We found a significant increase in the recording of recovery-oriented rehabilitation language following the intervention. The largest increase in use of a specific term was a 1.17% relative increase in the term ‘well-being’. The was followed by a 1.18% relative increase in use of the term ‘(consumer's name) will’, with an associated 1.90% relative decrease in the term ‘(consumer's name) was’ and an associated 0.69% relative decrease in the term ‘(consumer name) had’. These are significant outcomes, as ‘(consumer name) will’ is mostly used in the care review summaries to describe actions that the consumer intends to take to work toward recovery goals, whereas ‘(consumer name) was’ and ‘(consumer name) had’ is language that is backward-looking. Together, these results suggest a shift from language focusing on a consumer's past to language that describes actions that the consumer intends to take to work toward recovery goals. By contrast, we did not find significant changes in person-centred, non-recovery-oriented rehabilitation language, nor pejorative language. The lack of significant change in pejorative language can be explained by a floor effect attributable to extremely low rates of pejorative language before the intervention. Similarly, there was a relatively high rate of person-centred language before the intervention, creating a ceiling effect and likely explaining the lack of significant difference.

International studies have shown that recovery-oriented interventions have significant economic advantages. These include decreases to direct costs such as costs related to hospital stays and community treatment, as well as indirect benefits such as increased productivity.^16–18^ A multicentre study of mental health services in Ireland showed that those who received rehabilitation services were eight times more likely to remain in the community or live in less supported accommodation compared with those in receipt of standard care; those who received rehabilitation services were also more likely to show improvement in social functioning.^19^

Language can promote a sense of hope and empowerment in not only the person, but other stakeholders in the person's life, including clinicians, and can contribute positively to improved care for the person as a result.^1–4^ The type of language adopted by mental health services across verbal and written modalities has been shown to be a significant mediator of the behaviours of people with mental health concerns and those involved in their daily lives, as well as the attitudes and behaviours of mental health clinicians. Use of recovery-oriented language has, in turn, been shown to be associated with more frequent rehabilitation-focused behaviours and interventions by clinicians.^3^ This study has built upon a growing area of research into recovery and rehabilitation in mental health settings. The current study shows that with a structured intervention, clinicians can be guided to more accurately reflect the rehabilitation work undertaken as part of these interventions in their clinical documentation. In evaluating an intervention that promoted recovery-oriented rehabilitation and person-centred language in documentation, this study appears to be a first in addressing the gap in systematic research exploring the relationship between language and recovery-oriented practice in mental health settings identified by Kemp and Howard.^4^

This study also developed a method to objectively identify and evaluate the use of recovery-oriented rehabilitation language in documentation, which can be used by services to evaluate service provision and by clinicians to demonstrate professional knowledge and skills. As far as we are aware, no similar methodology has been used for a similar application, nor have we seen published studies that specifically classify and quantify recovery-oriented rehabilitation language in this way. This study also appears to be the first to develop a method to objectively identify and evaluate the use of recovery-oriented rehabilitation and person-centred language in documentation, and to begin generating a dictionary of such terms. This set of techniques, and the development of a dictionary of recovery-oriented rehabilitation terms, could have broader applications in mental health services evaluations; for example, in providing a means to automate auditing and evaluation of large volumes of clinical documentation that services accumulate. It could also be used by mental health clinicians to demonstrate professional knowledge and skill in the area of recovery-oriented language.

### Implications for practice

The implications of this study are that a structured training programme involving both group and individual interventions, and including instruction on formulation and rehabilitation principles, can lead to increased documentation of language that is recovery-oriented and rehabilitation-focused. Increased use of recovery-oriented rehabilitation language in documentation will then more adequately and appropriately reflect any rehabilitation work undertaken. Furthermore, use of recovery-oriented rehabilitation language has been hypothesised to lead to increased rehabilitation-focused practices and interventions by clinicians, and therefore more positive and recovery-focused experiences for consumers of mental health services.^3^ As such, the training programme used in this study, or one like it, could be offered to mental health clinicians wishing to increase their knowledge of rehabilitation in mental health settings and appropriately document rehabilitation-focused interventions, or to mental health services interested in increasing their overall ability to provide, document and evaluate rehabilitation-focused services. The change in documentation and the hypothesised increase in engagement in recovery-oriented rehabilitation practices could then lead to more positive, recovery-oriented outcomes for consumers, such as decreased hospital admissions, increased productivity and increased social functioning.

In addition to the training programme promoting an increased rehabilitation focus in documentation and outcomes, the second implication of this study was the development of a unique textual analysis methodology. One advantage of this textual analysis methodology is that, when paired with a suitable dictionary of terms, language in electronic medical records can be automatically audited for person-centred and rehabilitation-oriented language, and other types of language at scale.

### Limitations

It would be important to replicate the results of this study with a larger, more diverse clinician population. Further research in larger-scale, more diverse mental health services that evaluate the utility of the analysis methods used and add to the dictionary formed in this study would further contribute to developing efficient, automated means of evaluating documentation and services in mental health settings. Furthermore, future research using non-automatic, qualitative content analysis to validate the automated analysis would be beneficial. It would be prudent to check that the findings of the automated analysis align with what would emerge under a more traditional approach. This would more soundly justify the use of the automated process as a quick tool for services to track their rehabilitation language. Also, although the text analysis approach developed in this study could be used by services to evaluate service provision and by clinicians to demonstrate professional knowledge and skills, including recovery-oriented rehabilitation practice, this needs to be tempered by the fact that what clinicians write may be very different from what they do, and might not adequately capture verbal responses and clinical interactions. Although potentially useful, the current approach is limited by the inherent nature of documentation, which may contain formulaic terminology and phraseology resulting from convention and content that is required administratively but is not specifically relevant to rehabilitation, or able to be meaningfully phrased with reference to person-centred care.

The focus of the current study on the documentation of recovery-oriented rehabilitation language has meant that attitudinal components of the clinicians to the recovery approach and how this can manifest in practice are yet to be examined. Therefore, it will be important to evaluate if providing training such as that evaluated in this study and/or increasing recovery-oriented rehabilitation language in documentation leads to a corresponding increase in recovery-oriented rehabilitation attitudes and practice for clinicians. Future research should also include studies of consumer experiences of the language clinicians use both verbally and in documentation before and after clinicians are trained in recovery-oriented rehabilitation language, their perception of the rehabilitation work undertaken with clinicians at these time points and the impact of this on their recovery journey. Following on from this, the involvement of patients and carers in the development of the dictionary and classification of language did not occur in this study, and should be considered in future research. This would complement Davidson's findings that the type of language adopted by mental health services was a mediator in the behaviours of consumers, carers and mental health clinicians.^3^

Finally, it is unclear if patient characteristics may have played a role in the language documented by clinicians in this study. Given its size, it was not possible to investigate this in the current study. Future research should investigate the relationship between patient characteristics and the documenting of recovery-oriented rehabilitation language by clinicians.

In conclusion, language used in documentation of rehabilitation-oriented services needs to reflect the nature of the interventions provided. We found that the training programme developed for this study promoted increased use of recovery-oriented rehabilitation language in the clinical documentation of a rehabilitation-oriented, assertive community treatment team. The methodology used in this study to automate the evaluation of clinical documentation in electronic medical records has the capacity to identify recovery-oriented rehabilitation terms quickly and objectively, assisting assessment of documentation fidelity.

This computer-driven, textual analysis method identified improvements in recovery-oriented rehabilitation language following training. Our study suggests that brief interventions can affect the language of clinical documentation, and that automated text analysis may represent a promising approach for rapidly assessing recovery-oriented rehabilitation language in mental health services.

## Data Availability

The data that support the findings of this study are available from the corresponding author, D.S., upon reasonable request.

## Supplementary material

For supplementary material accompanying this paper visit http://doi.org/10.1192/bjo.2023.14. click here to view supplementary material
