# Supplementary material for: Measuring recovery-oriented rehabilitation language in clinical documentation to enhance recovery-oriented practice
Source: BJPsych Open. 2023 Feb 15;9(2):e36. doi: 10.1192/bjo.2023.14 (PMC9970174; doi:10.1192/bjo.2023.14)
Supplement: Supplementary file 1 [file S2056472423000145sup001.docx]

Recovery-Oriented Rehabilitation Language: Helping Clinicians to Be More Rehabilitation-Focussed in Documentation and Novel Ways to Measure This.

Corresponding Author: Dan Siskind, d.siskind@uq.edu.au

Supplementary Materials

**Rehabilitation Oriented Language Evaluation (ROLE) Manual**

Two group training sessions are delivered to mental health practitioners to review and further increase knowledge of rehabilitation, formulation, and treatment planning using rehabilitation oriented language. This language is to be included in documentation of interventions, as well as case plans/ reviews. Documentation is important to ensure that language reflects the specialised work completed by the mental health practitioners with the consumer. Training occurs over two sessions of roughly 45 mins – 1 hour in a group setting. The presenter should use the PowerPoint presentation to deliver material. Training session 1 covers rehabilitation, formulation and treatment planning, and training session 2 covers documentation or rehabilitation and ROLE. Following this, individual interventions are conducted in a one-on-one format with mental health practitioners. The manual will be presented in a step-by-step format to assist in delivery of the interventions. As a whole, the intervention aims to increase rehab focused language in Care Review summaries.

Rehabilitation Oriented Language is based on recovery-oriented language. Recovery-oriented language focuses on language used when discussing the individual. The language is used to convey hope and optimism, and support recovery in patients with mental health disturbance. Recovery-oriented language includes language that is:

- Respectful
- Non-judgemental
- Clear and understandable
- Free of jargon
- Carrying a sense of commitment, hope, and recovery

This type of language should not only be used with the consumer, but also in discussions with other mental health practitioners, and in documentation. We need to consider how our language might be read/ heard by the consumer, and consider what language may be beneficial in improving health and wellbeing. Specific examples of reframing language to recovery-oriented language can be found in Appendix A.

The following training sessions aim to increase awareness of recovery-orientated language, and to support mental health practitioners in implementation of this language in Consumer Care Review Summary and Plan (CCRSP) documentation.

**INTERVENTION TRAINING**

**Training Session 1**

The first training session covers use of recovery-oriented language in rehabilitation, formulation, and treatment planning. The training session is presented in approximately 30 minutes to mental health practitioners.

Materials required:

- Training session 1 PowerPoint presentation
- Copies of the following resources for each mental health practitioner:
  - *Is diagnosis enough to guide interventions in mental health? Using case formulation in clinical practice* paper: Macneil, Hasty, Conus, and Berk (2012)
    - Provided to support rationale for using formulation in CCRSP documentation
  - *Guide to appropriate language in mental health services*: NSW Government
    - Provided to support understanding and provide examples of recovery-oriented language
  - Mobile Intensive Rehabilitation Team (MIRT) Case Review Template
    - Provided as an example to formatting case reviews
  - Feedback form for Training Session 1
    - For mental health practitioners to provide feedback regarding the presentation and content

**Presentation**

**Part A: Recovery and Rehabilitation**

*Include the following points when presenting to mental health practitioners (in conjunction with the PowerPoint presentation):*

- Using formulation in care reviews is not a CAPA principle, but is related
- ROLE originates in needing to document the rehabilitation/ recovery work that is being completed by mental health practitioners with consumers. Some aspects of care that impact recovery/ rehabilitation were being overlooked and not included in documentation (e.g., symptomatology and cognition were not initially included, but need to be included to direct rehabilitation work). As such, formulation needs to be clear in documentation and reflect the work that is being done
- **GROUP DISCUSSION**: ask the group
  - What is recovery vs rehabilitation?
    - The group might identify that recovery is a journey and gives hope, recovery means returning to function and society. Remission from symptoms not enough, functional recovery is also important
    - Rehabilitation is the service provided by mental health practitioners to achieve recovery
- Review the definitions of recovery and rehabilitation on the slides:
  - “Recovery means gaining a sense of meaning, a positive identity, fulfilling relationships, the role of citizen and community member, the capacity to cope with adversity and recognition of the gifts and lessons learned through the recovery struggle” (PRA, 2002)
  - “Rehabilitation refers to the development of skills and supports needed to achieve one’s goals … focuses on increasing ability and builds on a person’s strengths to facilitate success in meeting the person’s own goals… Rehabilitation promotes a partnership” (PRA, 2002)
- Why rehabilitation is important:
  - Economic benefits including employment, economic savings (i.e., keeping out of hospital, relative costs of schizophrenia)
  - Rehabilitation is not diagnoses or treatment/ symptom reduction, it reflects functional improvement in relation to the consumers recovery goals

*Make sure the group understands, and answer any questions before moving to Part B.*

**Part B: Formulation**

The importance of formulation and applying recovery-oriented language to formulations in CCRSP documentation.

- Formulation is “the bridge between assessment and intervention” (Macneil, Hasty, Conus, & Berk, 2012). It serves a number of functions: understanding etiological factors, identifying key difficulties, guiding which interventions should be utilised and in what order, anticipating challenges in rehabilitation and identifying strengths and ways to overcome these
- Importantly, formulation is collaborative, immediate, and ongoing
- The key aspects that are important in formulation are: identifying the current problem (problem), and the factors that maintain the problem (perpetuating factors). The maintaining factors can be targeted in treatment to manage the problem. These two factors (problem and perpetuating factors) make up the 5 “P” model of formulation (alongside predisposing, precipitating, and protective factors)
- Problem: *identify what the problem is, i.e., the difficulties the consumer is experiencing. For example, not attending to ADLs*
  - It is important to consider one problem (identified by the consumer) and one treatment approach, where mental health practitioners are assigned a role and work together for efficiency to overcome the problem. In this case, the consumer is the “driver” (pit-stop metaphor)
  - To identify the problem: the problem is observable and measurable, and usually functional. The problem is not diagnosis or symptoms. If it is difficult to identify the problem, consider: if we videotaped the consumer over time, what do we see that is different at the end?
    - Tools that can be used to identify the problem: MIRT case review template, consumer goals, outcome measures, magic wand metaphor, consider what specifically you are doing with the consumer and what this is addressing
- Perpetuating: *what is keeping the problem going?*
  - Perpetuating factors are important because we want to measure and target these to improve the problem
  - Some common perpetuating factors that could be considered include:
    - Consumers experience of symptoms
    - Ongoing substance use
    - Behavioural patterns (e.g., avoidance, safety behaviours, withdrawal)
    - Biological patterns (e.g., insomnia, changes in appetite)
    - Cognitive difficulties
    - Skills deficits
    - Difficulties in understanding of support network
    - Reinforcers/ secondary gains
  - Tools to identify perpetuating factors: MIRT case review template, outcome measures, spiders web, discipline “checklist”, above list of examples
- **GROUP DISCUSSION**: ask the group
  - Identify a problem that a consumer is experiencing and consider how the problem was identified? What are the perpetuating factors? How were these factors identified?

The other 3 “P”s of the formulation model are also important to consider in formulation:

- Predisposing: *factors that may put a person at risk of experiencing their current problem*
  - Some common predisposing factors include:
    - Genetic factors (family history)
    - Other biological factors (e.g., brain injury, developmental difficulties)
    - Environmental factors (e.g., socio-economic status, trauma, modelling)
    - Psychological factors (e.g., maladaptive core beliefs, personality factors)
- Precipitating: *what started the problem? What was happening at the time when the consumer became unwell?*
  - These factors may have triggered the problem to occur
- Protective/ prognosis: *what are the person’s strengths? How likely is it that their recovery goals will be reached?*
  - Consider the factors that support the consumer towards achieving their recovery goals
- Rehabilitation planning follows from formulation, addresses perpetuating factors, and breaks down these factors into sub-tasks to be achieved
  - It is important to know outcomes and timeframes for each task, consider this in CCRSP documentation

*Make sure the group understands, and answer any questions. Remind mental health practitioners to complete the feedback form at the end of the training session.*

**Training Session 2**

The second training session covers documentation of recovery-oriented language and rehabilitation. The training session is presented in approximately 30 minutes to mental health practitioners.

- Materials required:
  - Training session 2 PowerPoint presentation
  - Copies of the following resources for each mental health practitioner:
    - Recovery Oriented System Indicators (ROSI)
    - Recovery Self-Assessment (RSA)
    - Recovery and Independent Living PEG Advisory Paper 9
    - Feedback form for Training Session 1

**Presentation**

**Part A: ROLE and Rehabilitation**

Outline that Training Session 2 will cover how to document the concepts outlined in Training Session 1 using rehabilitation oriented language.

- Recap information from Training Session 1, including:
  - The importance of including recovery and rehabilitation language in case reviews
  - Using the 5 “P”s model of formulation collaboratively to assist with treatment planning (partnership phase of CAPA)
  - The importance of rehabilitation oriented language in helping to convey the work that is being done beyond treatment and symptom reduction, and helps to convey hope (consider that how language is read/ heard by the consumer could positively contribute to their health and wellbeing)
- **PERSONAL REFLECTION**: ask the group to consider
  - Why might ROLE be a good idea?
    - The group might identify that it could be important to track rehabilitation, to communicate to others, to ensue hope, to represent your discipline, and to reflect the work completed with the consumer that may be different to acute teams
  - Why would not using ROLE be a bad idea?
  - How important is it to do this on a scale from 0-10?
- Outline the purpose of including ROLE in care reviews:
  - To remind the reader of recovery goals
  - To consider and outline rehabilitation work to come
  - Use in formulation and treatment planning
  - Summary of rehabilitation for that care period, plan for upcoming care period/ “letting go” (or graduation), not meant to be detailed history
- **GROUP DISCUSSION**: ask the group to consider the audience they are writing for
  - Who are you writing for, and what do they want to know?
    - The group might identify that there are many people that may read case reviews, including:
      - Intended audiences (the consumer, carer/family/friends, GP, NGO, other teams/services)
      - Unintended audiences (executive, funding bodies (NDIS), medico-legal, yourself)

*Make sure the group understands, and answer any questions before moving to Part B.*

**Part B: Documentation**

It is important to accurately document the work completed with the consumer and the treatment plan.

- When documenting rehabilitation work, consider:
  - What skills did the consumer develop?
  - What supports were put in place/ enhanced?
  - How are the skills and supports working together to aid recovery?
  - How have abilities increased?
  - What partnerships were formed/ quality of partnerships
  - What core/ specific partnership tasks were undertaken?
  - What were the outcomes measures that were used?
- When documenting treatment planning, consider:
  - Formulation
  - What are the steps for the next care period?
  - What core/ specific partnership tasks are you hoping to achieve?
  - What changes in outcome measures are you expecting?
  - *When entering the “letting go” (or graduation) phase, consider the overall plan, and final outcomes*
- Other useful tools for documenting rehabilitation are included in the handouts:
  - Recovery Oriented System Indicators (ROSI)
  - Recovery Self-Assessment (RSA)
    - To use on self and service as a prompt
  - Recovery Oriented Practice Index
  - Recovery Promotion Fidelity Scale
- Person-centred language is also important in ROLE
  - Person-centred language means that the individual is at the centre of service delivery, and language should reflect unconditional positive regard
  - Person-centred language needs to be respectful, non-judgemental, clear and understandable, free of jargon/ confusing data and speculation, carrying a sense of commitment, hope, and presenting the potential for opportunity
  - It conveys thoughts, feelings, facts and information, but also asks questions such as:
    - What else am I saying about the person?
    - How will someone else read this?
    - Do I give a sense of commitment, hope and present opportunity?
    - Do I convey an awareness and expectation of recovery?
  - Examples of using person centred language can be found in the “*Guide to appropriate language in mental health services*” (NSW Government) handout provided in Training Session 1

*Include a summary to reinforce the key points from the training sessions:*

- Rehabilitation-oriented and person-centred writing focuses on the person’s engagement and progress with their functional recovery
- It includes formulation, rehabilitation, and treatment planning in specific sections of the care review summaries

*Make sure the group understands, and answer any questions. Remind mental health practitioners to complete the feedback form at the end of the training session.*

**Individual Intervention**

The individual intervention component of the program was designed to provide one-on-one support to the mental health practitioners and their specific formulations and documentation. This also provides a time for mental health practitioners to ask questions and refine formulation and recovery-oriented language skills. The individual intervention should ideally be completed face-to-face, and the mental health practitioner should have an example of a case review to discuss.

During individual interventions, it is important to address the following points with the mental health practitioner:

- Have they used the P’s formulation, and in particular, clearly identified the presenting problem, and the perpetuating factors?
  - Ensure a clear *presenting problem* is identified, and ideally only 1-2 presenting problems should be identified. Presenting problems could include (1) difficulties maintaining self-cares, (2) difficulties engaging in meaningful activities. See *Training Session 1, Part B: Formulation* for more examples. Ensure that patient-centred language is used throughout, for example, identifying the problem as “the consumer experiences difficulties maintaining their self-care” as compared to “the patient cannot care for themselves”.
  - Ensure clear *perpetuating factors* are identified that are directly related to the presenting problem, i.e., the factors that keep the problem going. These may include (1) difficulties with motivation, (2) presence of delusional beliefs, (3) lack of social support. See *Training Session 1, Part B: Formulation* for more examples.
  - Ensure inclusion of some predisposing, precipitating, and protective factors in the documentation for further detail of the consumer.
- Have they outlined the work completed with the consumer during this care period, using rehabilitation-oriented language? This may include (1) providing psychoeducation, (2) forming partnerships with external agencies, (3) implementing environmental modifications. Consider asking the mental health practitioner:
  - What skills did the consumer develop?
  - What supports were put in place/ enhanced?
  - How are the skills and supports working together to aid recovery?
  - How have abilities increased?
  - What partnerships were formed/ quality of partnerships?
  - What core/ specific partnership tasks were undertaken?
  - What were the outcomes measures that were used?
- Have they outlined the plan, using rehabilitation-oriented language, for the upcoming care period/ “letting go” (or graduation)? Consider asking the mental health practitioner:
  - What core/ specific partnership tasks are you hoping to achieve?
- Have they used patient-centred language throughout? Refer to Appendix, *Guide to appropriate language in mental health services (NSW Government)* for rephrasing into patient-centred language.
- Ensure the mental health practitioner has the opportunity to ask questions.

**Rehabilitation Oriented Language Evaluation (ROLE) Intervention PowerPoint Presentation**

*Supplementary Table 1 Final terms included in the pilot dictionary organised by category*

| **Category** | **Terms** |
| --- | --- |
| **Recovery-oriented rehabilitation** | support, family, social, continue, CNAME will, work, recovery, goals, routine, will, factors, rehab, strategies, difficulties, able, independently, healthy, prompting, maintain, maintaining, plan, domestic, supports, skills, associated with, rehabilitation, vocational, coping, outcomes, letting go, independent, participate, cooking, lifestyle, group, goal, improved, in next, made, CAPA, encourage, mindfulness, wellbeing, engaged, leisure, opportunities, into practice, attends, challenges, relaxation, supportive, impact, identified, improvement, participating, completing, building, demonstrated, network, functioning, motivated, establish, progress, plans, swimming, partnership, engages, protective, meals, offered, feels, benefit, reinforcement, strong, encouragement, manages, metabolic monitoring, living skills, to practice, care plan, choices, was able, perpetuating, expressed, connections, clean, achieving, pursue, achieved, achieve, maintaining mental, participated, care in partnership |
| **Person-centred** | anxiety, CNAME was, due to, well, reports, continues to, attend, limited, unwell, understanding, predisposed, CNAME had, enduring, difficult, engaging, difficulty, establishing, managing, believes, suffers, tends, hasn't been able, struggles, inability, limited social, CNAME requires, signs, not been able, is not able |
| **Pejorative** | absconding, failed, delusional, non-compliant, non-compliance, substance abuse, bizarre |

*CNAME = consumer name, de-identified

*Supplementary Table 2 Care Review Summary headings*

| **Headings** | **Subheadings/Instructions** |
| --- | --- |
| Treatment Status | Mental Health Act 2016 status  Conditions of MHA order  Other status |
| Substitute Decision Maker | Substitute Decision Maker  Advanced Health Directive  Enduring Power of Attorney  Guardian  Administrator |
| Consumer receiving treatment from | Team name  Type of case review |
| Diagnoses and Principal Drug of Concern | ICD10 code  Diagnosis |
| Date Risk Screen completed |  |
| Formulation | Summarise key issues from the assessment and provide your clinical impression. Requires a longitudinal perspective which examines the consumer’s historical, contextual and current factors. It should consider co-occurring conditions and the relationship between, and impact of mental illness, substance use and addictive behaviours, and risk factors |
| Summary of care and treatment provided since last assessment/review | Include consumer’s response to treatment and risk mitigation strategies, their engagement with treatment, physical health and medications ceased. Update collateral obtained/still to be obtained since last assessment/review |
| Medications |  |
| Multidisciplinary Team Discussion | Summarise the current issues for the consumer and the key points that were considered by the multidisciplinary team |
| Treatment Recommendations | Summarise recommendations made by the multidisciplinary team for the consumer's treatment. Detailed actions should be incorporated in the Care Plan |
| Senior clinical lead name |  |
| Key contributors to the case review | All persons present internal and external to the treating team. Provide the name, role/discipline and contact number/agency name for each person. |
| Distribution list | External to Queensland Health. A copy of this form has been provided to (please tick):   - Consumer - Family/carer - GP - Private Practitioner - Other |
